# Metabolic Abnormalities in Patients with Chronic Disorders of Consciousness

**DOI:** 10.14336/AD.2020.0812

**Published:** 2021-04-01

**Authors:** Jie Yu, Fanxia Meng, Fangping He, Fei Chen, Wangxiao Bao, Yamei Yu, Jintao Zhou, Jian Gao, Jingqi Li, Yao Yao, Woo-ping Ge, Benyan Luo

**Affiliations:** ^1^Department of Neurology and Brain Medical Center, First Affiliated Hospital, School of Medicine, Zhejiang University, Hangzhou 310003, China.; ^2^Children's Research Institute, Department of Neuroscience, University of Texas, Southwestern Medical Center, Dallas, TX 75390, USA.; ^3^Department of Rehabilitation, Hangzhou Hospital of Zhejiang Armed Police Corps, Hangzhou 310051, China.; ^4^Department of Pharmaceutical and Biomedical Sciences, University of Georgia, GA 30602, USA.; ^5^Chinese Institute for Brain Research, Beijing 102206, China.

**Keywords:** chronic disorders of consciousness, targeted metabolomics, lipidomics, diagnosis, biomarkers, minimally conscious state (MCS), vegetative state (VS), Alzheimer's disease (AD)

## Abstract

The vegetative state (VS) and minimally conscious state (MCS) are two major types of chronic disorders of consciousness (DoC). The assessment of these two consciousness states generally relies on the Coma Recovery Scale-Revised (CRS-R) score, but a high misdiagnosis rate limits the generalized use of this score. To identify metabolites in human plasma that can accurately distinguish VS from MCS patients, comprehensive plasma metabolic profiles were obtained with targeted metabolomics analysis and untargeted and targeted lipidomics analysis. Univariate and multivariate analyses were used to assess the significance of differences. Compared with healthy controls (HCs), the DoC groups, Emerged from Minimally Conscious State (EMCS) group and Alzheimer’s disease (AD) group had significantly different metabolic profiles. Purine metabolism pathway differed the most between the DoC (MCS and VS) and HC groups. In this pathway, adenosine, ADP, and AMP, which are the derived products of ATP degradation, were decreased in the MCS and VS groups compared to healthy controls. More importantly, we identified certain lipids for which the levels were enriched in the VS or MCS groups. Specifically, phosphatidylcholine, (38:5)-H (PC(38:5)-H), and arachidonic acid (AA) differed substantially between the VS and MCS groups and may be used to distinguish these two groups of patients. Together, our findings suggest that metabolic profiling is significantly altered in patients with chronic DoC.

There are two major different groups of patients with chronic disorders of consciousness [1]: the vegetative state (VS) and minimally conscious state (MCS) [2, 3]. Patients in a VS are awake but unaware of themselves or their environment [4], but MCS patients have reproducible signs of awareness and exhibit fluctuations in consciousness [5]. Generally, there are two outcomes for these patients: one is a permanent VS, which refers to patients who cannot be aroused after 1 year and eventually die, and the other is progression to MCS with a gradual increase in independence [6]. Therefore, an accurate and reliable diagnosis of VS or MCS in the early stage can greatly benefit therapy and recovery. However, the assessment of these two consciousness states in clinics largely relies on the Coma Recovery Scale-Revised (CRS-R) score, but high misdiagnosis rate limits the generalized use of this score.

Metabolites are the most abundant biomolecules in the human body, and a global metabolic profile can serve as a direct indicator of the metabolic changes in a biological system [7]. Although impermeable to metabolites under normal conditions, the blood-brain barrier becomes disrupted under pathological conditions [8] such as neuroinflammation and traumatic brain injury. A compromised blood-brain barrier allows the exchange of metabolites between the brain and the plasma, meaning that the plasma metabolic profile can reflect metabolism in the brain and, to some extent, brain disorders. Recent studies have shown that abnormal metabolism is associated with several diseases, including stroke [9], diabetes [10] and a number of cancers [11-13]. Indeed, altered metabolism can participate in the onset of inflammation, apoptosis, and gene expression after severe brain injury [14-17]. Furthermore, numerous studies have demonstrated that lipids may serve as potential biomarkers for certain disorders, including those of the brain [18, 19]. Therefore, understanding brain and plasma metabolic profiles under both normal and pathological conditions is of importance and may lead to the identification of biomarkers for certain diseases.

Metabolomics and lipidomics are high-throughput techniques for the systematic analysis of a comprehensive metabolic profiling. They can effectively detect changes in lipid classes and even particular lipid species and predict their potential mechanisms and functions in various biological processes [20]. However, the application of metabolomics and lipidomics to the understanding of the pathogenesis of DoC is rare. To address this knowledge gap, we examined both metabolite and lipid changes specific to different levels of consciousness with the ultimate goal of exploring the potential mechanisms of DoC. We also did comparison of DoC patients with Alzheimer's disease (AD) and Emerged from Minimally Conscious State (EMCS) in metabolic profiling. Using both untargeted and targeted lipidomic analyses, we not only identified potential biomarkers that can distinguish between VS and MCS patients but also assessed the specificity and sensitivity of those biomarkers.

## MATERIALS AND METHODS

### Ethics statement

Written informed consent was obtained from each patient’s legal guardian and the healthy volunteers (Number:2015310). This study was approved by the Ethics Committee of the First Affiliated Hospital, School of Medicine, Zhejiang University, and Hangzhou Hospital of Zhejiang Armed Police Corps, China.

### Subjects

Patients with chronic DoC and EMCS after severe traumatic brain injury (TBI) were recruited from the rehabilitation units of Hangzhou Hospital of Zhejiang Armed Police Corps, China at the time of their admission. The causes of brain damage of all of the patients included in this study were traffic accidents and falls (see details of each patient in Supplementary Table 5). Glasgow Coma Scale was used for assessment of the severity of brain injury in acute stage. Diagnosis of VS, MCS and EMCS were based on 5 assessments within 10 days by DoC experts from Hangzhou Hospital of Zhejiang Armed Police Corps using the Coma Recovery Scale-Revised (CRS-R) [5]. In brief, these states are defined as follows. VS: patients can open their eyes and preserve sleep-wake cycles, but are unaware of themselves and their surroundings; MCS: patients have reproducible signs of awareness and exhibit fluctuations in consciousness; EMCS: recovery of functional object uses or communication after a chronic disorder of consciousness. All samples were collected from patients that maintained these physiological states (VS, MCS, EMCS) for at least 1 month. Patients with AD were recruited from the memory clinic of the First Affiliated Hospital of Zhejiang University. AD patients in this study were diagnosed according to both the criteria of the Diagnostic and Statistical Manual (DSM)-IV [21] and guidelines of the National Institute of Neurological and Communicative Disorders and the Stroke and Alzheimer Disease and Related Disorders Association (NINCDS-ADRDA) [22]. Age- and gender-matched healthy controls (HCs) were recruited from the First Affiliated Hospital, School of Medicine, Zhejiang University.

Subjects comprised three groups for targeted metabolomic analysis: 8 HCs (average age 55.4 ± 4.2 years; 3 males and 5 females); 12 VS patients (average age 63.8 ± 9.6 years; 9 males and 3 females) and 11 MCS patients (average age 63.2 ± 7.9 years; 6 males and 5 females). Subjects for targeted and non-targeted lipidomics included 32 HCs (average age 48.3 ± 14.2 years; 23 males and 9 females); 32 VS patients (average age 48.4 ± 15.4 years; 25 males and 7 females) and 22 MCS patients (average age 52.2 ± 12.5 years; 12 males and 10 females). Further information is shown in Table 1.

Subjects comprised another three groups for targeted metabolomic analysis: 6 HCs (average age 55.8 ± 4.8 year; 2 males and 4 females); 15 EMCS patients (average age 55.1 ± 8.7 years; 10 males and 5 females); 19 AD (average age 70.5 ± 8.3 years; 11 males and 8 females). Further information is shown in Supplementary Table 1.

All subjects were categorized based on their Glasgow Coma Scale and CRS-R scores. Centrally acting drugs, neuromuscular function blockers and sedation were discontinued for at least 24 h before blood samples were drawn. For all patients, the CRS-R, Glasgow Coma Scale were determined at the time of admission. Patients with an acute infectious disease, liver dysfunction, or kidney dysfunction were excluded from the study. We also excluded those patients who received a special treatment, such as transcranial magnetic stimulation (TMS) or transcranial direct current stimulation (tDCS).

**Table 1:** Clinical characteristics of chronic DoC patients and HCs.

		Metabonomics				Lipidomics		
Characteristics	HCs	VS	MCS	*P* value	HCs	VS	MCS	*P* value
Patients (n)	8	12	11	/	32	32	22	/
Male/Female (n)	3/5	9/3	6/5	0.157	23/9	25/7	12/10	0.170
Age (years)	55.4 ± 4.2	63.8 ± 9.6	63.2 ± 7.9	0.323	48.3 ± 14.2	48.4 ± 15.4	52.2 ± 12.5	0.575
GCS	/	6.8 ± 1.3	8.8 ± 1.5	0.003	/	7.8 ± 1.2	10.8 ± 1.8	0.413
CRS-R	/	5.1 ± 1.3	10.6 ± 5.7	<0.001	/	4.2 ± 1.8	13.1±4.6	<0.001
Cause	/	TBI	TBI	/	/	TBI	TBI	/

Continuous variables are expressed as the mean ± standard deviation (SD); TBI: traumatic brain injury; GCS: Glasgow Coma Scale; CRS-R: Coma Recovery Scale-Revised scores; DoC: disorders of consciousness; HCs: healthy controls; VS: vegetative state; MCS: minimally conscious state.

### Blood sample collection

Fasting blood samples (5 mL) were collected in vacutainer tubes containing EDTA serum separator tubes (BD SST^TM^ II Advance, Loughborough, UK using standardized phlebotomy procedures. The samples were immediately placed on ice for 15 min and then centrifuged in the laboratory (plasma: 5 min, 1000 × g, 4 °C; serum: 15 min, 3000 rpm, 4 °C). After confirming that hemolysis had not occurred, three 100-µL plasma samples were taken from each tube for metabolomics reserves and metabolomics experiments as described [13]. Care was taken to ensure that only plasma was collected from each centrifuged sample. All samples were stored at -80 °C.

### Purification of lipids and metabolites from blood

Serum samples were thawed at 4 °C, and 100-µL aliquots were mixed with 240 µL cold methanol and 200 µL cold water to precipitate protein. Then, 800 µL methyl tert-butyl ether (MTBE) was added, and the samples were mixed and maintained at room temperature for 20 min. Then centrifuged and removed the supernatant. The mixtures were centrifuged for 15 min (8000 × *g*, 10 °C), and the upper/organic phase was removed and dried under a nitrogen stream. For liquid chromatography-coupled mass spectrometry (LC-MS) analysis, the samples were redissolved in 200 µL isopropanol with vigorous mixing. To monitor the stability and repeatability of LC-MS, quality-control samples were prepared by pooling 10 µL of each sample, and these were analyzed together with the other samples. A quality-control sample was analyzed after every 8 samples. Metabolite from plasma for targeted metabolomics analysis was extracted as described in our previous publicaiton [13].

### UHPLC-MRM-MS/MS analysis

Metabolites were reconstituted in 50 µL of 0.03% formic acid in water, vortex-mixed, and centrifuged to remove debris. Samples were then injected in randomized order onto a SCIEX QTRAP 5500 liquid chromatograph / triple quadrupole mass spectrometer. Separation was achieved on a Phenomenex Synergi Polar-RP HPLC column (150 × 2 mm, 4 µm, 80 Å) using a Nexera Ultra High-Performance Liquid Chromatograph (UHPLC) system (Shimadzu Corporation, Kyoto, Japan). The mobile phases employed were 0.03% formic acid in water (A) and 0.03% formic acid in acetonitrile (B). The column was maintained at 35°C and the samples were kept in the autosampler at 4°C. The flow rate was 0.5 mL/min, and injection volume 20 µL. The mass spectrometer was used with the electrospray ionization (ESI) source in multiple reaction monitoring (MRM) mode. The MRM MS/MS detector conditions were set as follows: curtain gas 30 psi; ion spray voltages 1200 V (positive) and -1500 V (negative); temperature 650°C; ion source gas 1 with 50 psi; ion source gas 2 with 50 psi; interface heater on; entrance potential 10 V. Dwell time for each transition was set at 3 msec. Samples were analyzed in a randomized order, and MRM data was acquired using Analyst 1.6.3 software.

### Untargeted lipidomics (LC-MS/MS)

Analysis was performed on a Q Exactive plus mass spectrometer (Thermo Scientific) that was coupled to UHPLC Nexera LC-30A (SHIMADZU) in both the positive and negative electrospray ionization modes, the LC mobile phase contained a 10 mM aqueous solution of ammonium acetonitrile formate in acetonitrile: water (6:4, buffer A) and 10 mM ammonium formate in acetonitrile: isopropanol (1:9 v/v, buffer B); The gradient was 30% B for 7 min and was linearly increased to 100% in 18 min and kept for 0.1 min. Finally, a 4.9 min of re-equilibration period was employed. The gradients were at a flow rate of 0.3 mL/min, and the column temperatures were kept constant at 45°. A 2 µL aliquot of each sample was injected. Full-scan spectra were collected in mass-to-charge ratio (m/z) ranges of 200-1800 and 250-1800 for positive- and negative-ion modes, respectively?

The mass-to-charge ratio of lipid molecules to lipid fragments was collected by the following method: after each full scan, 10 fragment patterns (MS2 scan, HCD) were collected.

### Medium- and long-chain fatty acid measurements

The serum samples were thawed on ice, and 50 µL of each sample was added into a 2-mL glass centrifuge tube. Then, 1 mL chloroform methanol (2:1 v/v) was added. After ultrasonication for 30 min, 2 mL of 1% sulfuric acid in methanol was added to the supernatant. The mixture was incubated in an 80 °C water bath for 30 min to achieve fatty-acid esterification for methyl esterification. Then, 1 mL n-hexane and 5 mL water were added, and vortex mixed. The supernatant (500 µL) was spiked with an internal standard (25 µL of 500ppm methyl salicylate), mixed, and subjected to GC-MS using an Agilent Model 7890A/5975C GC-MS system. To quantify medium- and long-chain fatty acid, Supelco 37-component FAME (fatty-acid methyl ester) mix (Sigma-Aldrich) was used to construct a calibration curve for the concentration range of 0.5-1000 mg/L. The IS was used to correct for injection variability between samples and minor changes in the instrument response.

The samples were separated with an Agilent DB-WAX capillary GC column (30 m × 0.25 mm ID × 0.25 µm). The initial temperature was 50 °C and remained as such for 3 min. The temperature was then increased to 220 °C at 10 °C/min, and remained at 220 °C for 20 min. The carrier gas was helium (1.0 mL/min). A quality-control sample was used for testing and evaluating the stability and repeatability of the system. The temperatures of the injection port and transmission line were 280 °C and 250 °C, respectively. The electron bombardment ionization source, SIM (Selected ion Monitor) scanning mode, and electron energy were 70 eV.

### Short-chain fatty acid measurements

The serum samples were thawed on ice, and 100 µL aliquots were added into a 2-mL glass centrifuge tube mixing with 50μL of water with 15% phosphoric acid and 150 µL of 5 µg/mL 4-methyl valeric acid (IS). The suspensions were homogenized for about 1 min and centrifuged for 10 min at 12000×g. 1μl supernatant was taken for GC-MS analysis using an Agilent Model 7890A/5975C GC-MS system. To quantify short-chain fatty acid, a calibration curve for the concentration range of 0.1-100 ug/ml was constructed. The IS was used to correct for injection variability between samples and minor changes in the instrument response.

The samples were separated with an Agilent HP-INNOWAX capillary GC column (30 m × 0.25 mm ID × 0.25 µm). The initial temperature was 90 °C and was increased to 120 °C at 10 °C/min, after which the temperature was increased to 150 °C at 5 °C/min and then to 250 °C at 25 °C/min, where it remained for 2 min. The carrier gas was helium (1.0 mL/min). The temperatures of the injection port and transmission line were 250 °C and 230 °C respectively. The electron bombardment ionization source, SIM (Selected ion Monitor) scanning mode, and electron energy were 70 eV.

### Data processing

Lipid identification (secondary identification), peak extraction, peak alignment, quantification, and other treatments were assessed with LipidSearch software version 4.1 (Thermo Scientific™). The main parameters were as follows: precursor tolerance, 5 ppm; product tolerance, 5 ppm; product ion threshold, 5%. Lipid molecules with a relative standard deviation of >30% were removed. For the data from LipidSearch, the lipid molecules with missing-group values >50% were removed. During GC-MS analysis, the peak area and retention time were determined with MSD ChemStation software. Then, a standard curve was drawn, and the concentrations of medium- and long-chain fatty acids in the samples were calculated.

### Pattern recognition analysis

After being normalized and integrated by using support vector regression, the processed data were uploaded into MetaboAnalyst for further analysis.

Principal component analysis (PCA) and orthogonal partial least squares-discriminant analysis (OPLS-DA) models were established using SIMCA-P 14.1 (Umetrics, Umea, Sweden), and these models were used to analyze data collected from both positive and negative models after log transformation and Pareto scaling. The variable importance in the projection (VIP) of each variable in the OPLS-DA model was calculated to determine its contribution to the classification. Univariate analysis included the Student's *t*-test and variable fold-change analysis. Hierarchical cluster analysis and correlation analysis were performed with R software.

**Figure 1.**
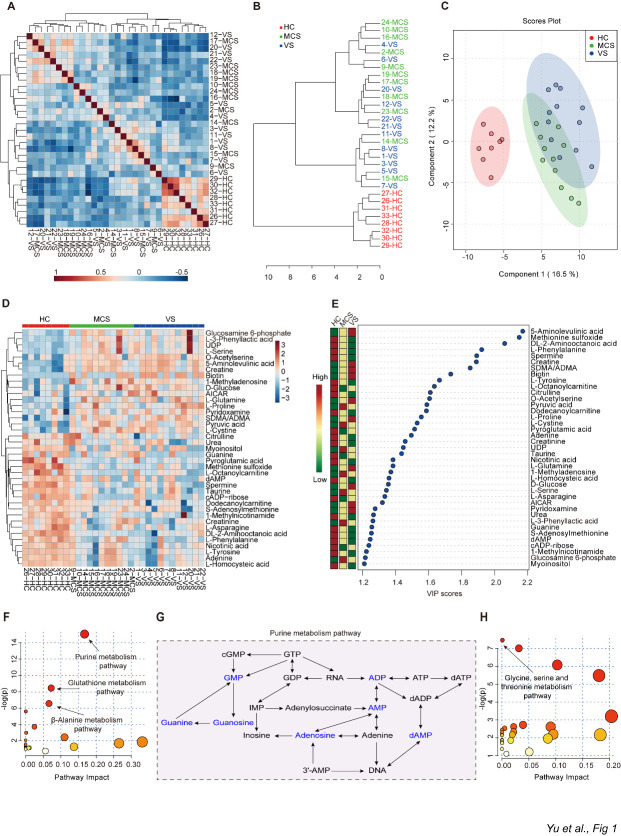
Metabolites responsible for distinguishing HC, MCS and VS groups. A) The heatmap of the correlation coefficients of the HC, MCS, and VS groups. B) Dendrogram of the three groups (HC, MCS, and VS) to show the clustering of all 31 samples. C) Principal component analysis of the plasma metabolome from three groups. D) Heatmap of the most abundant metabolites in three groups, as identified by VIP scores in PLS-DA. Each sample represents a single column. Red color indicates the greater abundance of metabolite. PLS-DA: partial least-squares discriminant analysis; VIP: variable importance in projection. E) Top 39 metabolites in plasma based on VIP scores for differentiating among HC, MCS, and VS groups. The colored boxes on the right indicate the relative concentrations of the corresponding metabolite in each group. F) A summary of pathway analysis using down-regulated metabolites in MCS and VS groups when compared to HC group. The size of the circle represents the pathway impact and the color represents the P value. Smaller P values and larger pathway impact indicate higher influential pathways. -log(*p*) = minus logarithm of the *P* value. G) A part of purine metabolism pathway in HC, MCS, group and VS groups. Metabolites in blue decreased in MCS group and VS group compared to HC group. H) A summary of pathway analysis using up-regulated metabolites in MCS group and VS group compared to HC group. HC: healthy controls; VS: vegetative state; MCS: minimally conscious state.

**Figure 2.**
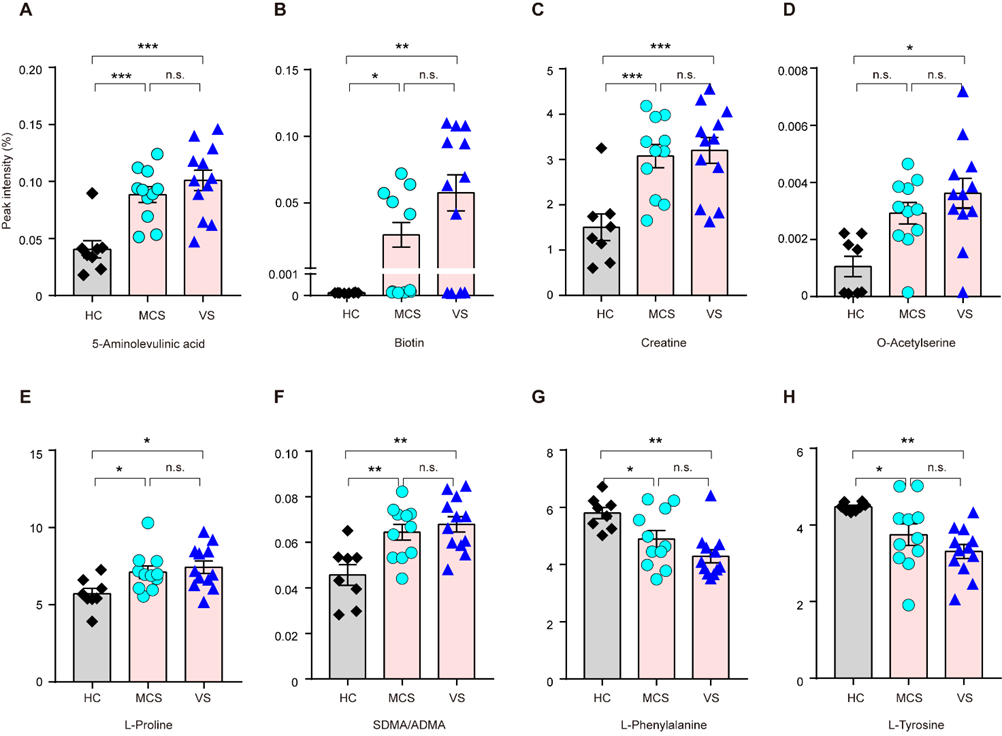
Identifying continuously up-regulated or down-regulated metabolites in HC, MCS, and VS groups. Among all metabolites that we analyzed, 5-aminolevulinic acid A), biotin B), creatine C), O-acetylserine D), L-proline E), and SDMA/ADMA F) gradually increased from HC, MCS to VS, whereas two metabolites, L-Phenylalanine G) and L-Tyrosine H) gradually decreased from HC, MCS to VS. Data represent as the mean ± SEM; *P<0.05, **P<0.01, ***P<0.001, n.s.: no significant difference, one-way ANOVA. SDMA: Symmetric dimethylarginine; ADMA: Asymmetric dimethylarginine. HC: healthy controls; VS: vegetative state; MCS: minimally conscious state.

### Statistical analysis

Metabolites and lipids with a variable importance in the projection value of >1 was further subjected to the Student’s t-test at the univariate level to measure the significance of each molecules. Statistical analyses were performed using SPSS version 18.0. Continuous variables are presented as the mean ± standard error of the mean (SEM). Unique lipids from each group were identified by decision trees from the panels of lipids and were subjected to leave-one-subject-out cross-validation. Receiver-operating characteristic (ROC) analyses and logistic regression were constructed to calculate the best cutoff point and area under the curve for the candidates. The statistical significance (*P*<0.05) of differences in metabolite levels was assessed using one-way analysis of variance (ANOVA) followed by post hoc Bonferroni, Mann-Whitney U, Kruskal-Wallis Test or Chi-square tests, where appropriate. Pearson linear correlation analysis was used to evaluate the association between lipids and CRS-R scores.

## RESULTS

In our study, 23 patients who suffered from severe TBI were recruited for targeted metabolomic study and an additional 54 TBI patients were recruited for lipidomic study (Supplementary Fig. 1). Another 40 participants, including HCs, EMCS patients and AD patients, were recruited for metabolomics. Clinical and demographic characteristics are presented in Table 1 and Supplementary Table 1. For some patients, we collected blood samples for the 2^nd^ or 3^rd^ time, if he/she was at different stages (VS, MCS).

**Figure 3.**
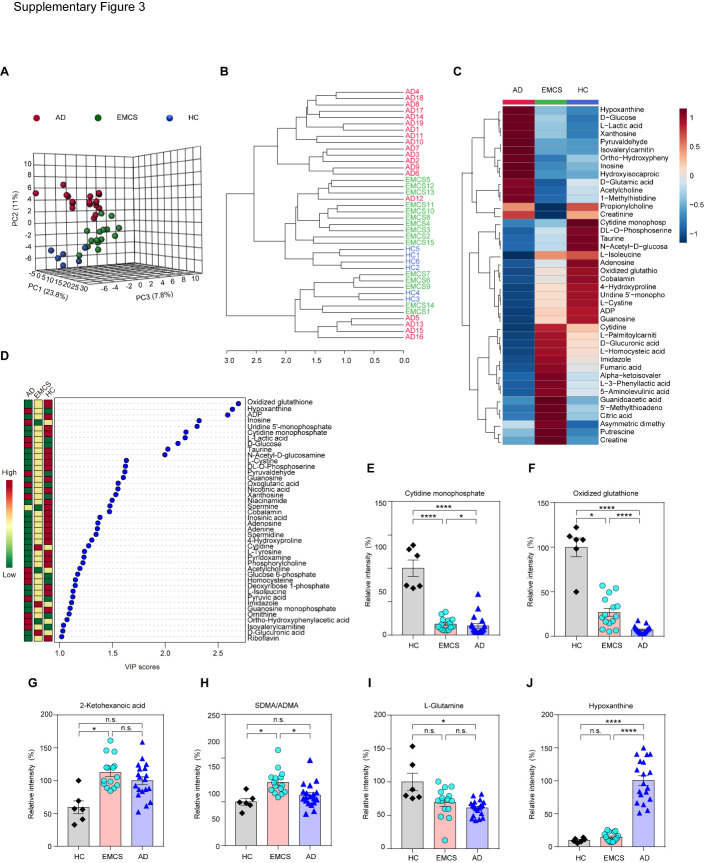
Metabolites responsible for distinguishing HC, EMCS and AD groups. A) A principal component analysis of the plasma metabolomes from three groups. B) Dendrogram of the three groups (HC, EMCS, and AD) showing the clustering of all 40 samples. C) Heatmap of the most abundant metabolites in the three groups, as identified by VIP scores. Each group represents a single column. Red color indicates greater abundance of the metabolite. VIP: variable importance in projection. D) Top 42 metabolites in plasma based on VIP scores for differentiating among HC, EMCS, and AD groups. The colored boxes on the right indicate the relative concentrations of the corresponding metabolite in each group. E-J) Difference analysis for Cytidine monophosphate, Oxidized glutathione, 2-Ketohexanoic acid, SDMA/ADMA, L-Glutamine and Hypoxanthine. Data are represented as mean ± SEM; *P<0.05, ****P<0.0001, n.s.: no significant difference. Student’s t-test. SDMA: Symmetric dimethylarginine; ADMA: Asymmetric dimethylarginine; HC: healthy control; AD: Alzheimer's disease; EMCS: Emerged from Minimally Conscious State.

### Targeted metabolomic analysis of plasma from VS and MCS patients and HCs

Comprehensive metabolomics measurement was performed on fasting plasma samples from the HC, MCS, and VS groups using UHPLC-MRM-MS/MS. In this study, a total of 137 distinct metabolites were reliably identified with our targeted metabolomic analysis. The 31 samples could be divided into HC, MCS, and VS groups via cluster analysis (Fig. 1A and B). To investigate the potential relationships among different samples in the three groups, we performed a correlation analysis and observed that there was a high correlation among the samples within each group (Fig. 1A), which is consistent with the results of our unsupervised PCA analysis: the samples from the MCS group were located between the HC and VS groups but were closer to those of the VS group (Fig. 1C). With partial least squares discriminant analysis (PLS-DA), we performed feature selection to identify metabolites that maximized separation between the MCS, VS, and HC groups. The importance of a metabolite in the model is measured by the Variable Importance in Projection (VIP) score, which is the weighted sum of squares of the PLS loadings for that variable. We identified 39 metabolites with a VIP score > 1.2 (Fig. 1D-E). Several metabolites, including 5-aminolevulinic acid, creatine, and biotin, showed a positive correlation with severity of brain injury: (MCS: L-cystine, pyruvic acid; VS: L-tyrosine, L-proline. Figure 1E). To further explore the metabolic pathways that potentially contribute to chronic disorders of consciousness, we carried out a global metabolic pathway analysis using the metabolites with high VIP scores (fold change >1.5) to compare the MCS and VS groups after PLS-DA. Among the 21 pathways that differed between MCS & HC and MCS &VS, the purine metabolism pathway, glutathione metabolism pathway, and β-Alanine metabolism pathway showed the most significant differences (Fig. 1F). In the purine metabolism pathway, adenosine, ADP, and AMP, which are the derived products of adenosine triphosphate (ATP) degradation, were decreased in the MCS and VS groups compared to healthy controls, indicating a dysfunction of purine metabolism in chronic disorders of consciousness (Fig. 1G). In addition, among the pathways up-regulated in the MCS and VS groups compared to the HC group, we observed that glycine, serine, and threonine metabolism pathways were the most significantly affected in patients with MCS and VS compared to healthy subjects (Fig. 1H and Supplementary Fig. 2).

**Figure 4.**
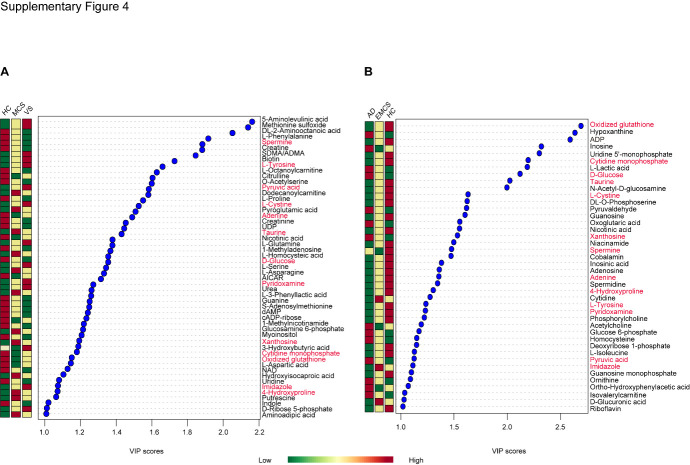
Top metabolites in plasma based on VIP scores for differentiating among HC, MCS, and VS groups; HC, EMCS, and AD groups. A) Top metabolites in plasma based on VIP scores >1 for differentiating among HC, MCS, and VS groups; B) Top metabolites in plasma based on VIP scores >1 for differentiating among HC, EMCS, and AD groups. The colored boxes on the right indicate the relative concentrations of the corresponding metabolite in each group The colored boxes on the right indicate the relative concentrations of the corresponding metabolite in each group. HC: healthy control; VS: vegetative state; MCS: minimally conscious state; AD: Alzheimer's disease; EMCS: Emerged from Minimally Conscious State.

**Figure 5.**
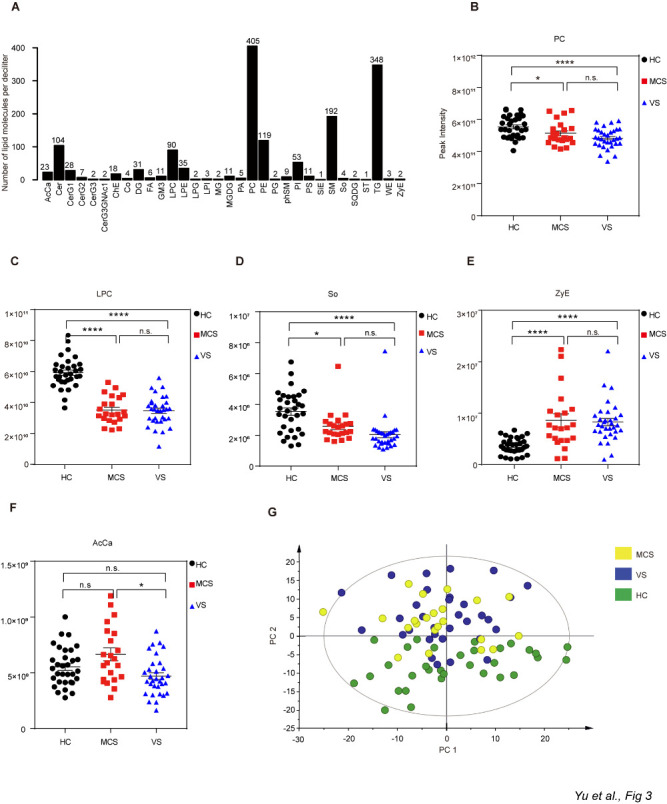
Preliminary analysis of lipids. A) Subclasses of serum lipids. B-F) Difference analysis for certain lipid subclasses between the VS and MCS groups and the HC group. Data represent as the mean ± SEM; *P<0.05, **P<0.01, ****P<0.0001, n.s.: no significant difference, one-way ANOVA. G) Principal component analysis (PCA) score plot. AcCa: acyl carnitine; PC: phosphatidylcholine; LPC: lysophosphatidylcholine; PE: phosphatidylethanolamine; LPE: lysophosphatidylethanolamine; PS: phosphatidylserine; PI: phosphatidylinositol; LPI: lysophosphatidylinositol; PG: phosphatidylglycerol; LPG: lysophosphatidylglycerol; PA: phosphatidic acid; Cer: ceramides; CerG1-G3, CerG3GNAc1: simple Glc series; ChE: cholesterol ester; Co: coenzyme; DG: diglyceride; FA: fatty acid; GM3: gangliosides; MG: monoglyceride; MGDG: monogalactosyldiacylglycerol; phSM: phytosphingosine; SiE: sitosterol ester; SM: sphingomyelin; So: sphingosine; SQDG: sulfoquinovosyldiacylglycerol; ST: sulfatide; TG: triglyceride; WE: wax exters; ZyE: zymosterol; HC: healthy controls; VS: vegetative state; MCS: minimally conscious state.

To further determine which metabolites are associated with consciousness, we compared the values of metabolites between HC & MCS, and between MCS & VS. VS patients have more severe brain damage than MCS patients, while healthy controls represent a population without known brain damage. The metabolites that show a gradual increase across HC, MCS, and VS (in that order) are likely the metabolites that are positively correlated with the severity of brain injury. Through paired comparison among three groups, we observed that 6 metabolites, 5-aminolevulinic acid, biotin, creatine, O-acetylserine, L-proline, and symmetric dimethylarginine/asymmetric dimethylarginine (SDMA/ADMA) increased, whereas two metabolites, L-Phenylalanine and L-Tyrosine, decreased in MCS and VS groups. All of these metabolites were significantly different in the VS and MCS groups compared to the HC group, but none of them showed significant difference between the MCS and VS groups (Fig. 2), suggesting that it is hard to distinguish the MCS and VS groups with these metabolites alone.

With unsupervised multivariate data analysis or cluster analysis, we found that samples from HC (6 healthy controls), EMCS, and AD patients could be easily distinguished (Fig. 3A and B), suggesting that metabolic abnormalities in patients with different neurological diseases differ from each other. We identified 42 metabolites with a VIP score > 1 in PLS-DA analysis (Fig. 3C-D). Compared to HCs, L-glutamine and hypoxanthine were significantly changed in plasma from AD patients in our study, which is consistent with previous studies [23, 24], but we did not observe this alteration in plasma of EMCS patients when compared to HCs (Fig. 3I-J). We found that 2-ketohexanoic acid and SDMA/ADMA were unique metabolites in EMCS patients (Fig. 3G-H). We observed that some metabolites like cytidine monophosphate, oxidized glutathione, SDMA/ADMA and hypoxanthine significantly differed between EMCS and AD patients, which strongly indicates that plasma metabolites differ significantly among these brain diseases, even though both involve neuronal degeneration (Fig. 3E-F).

**Figure 6.**
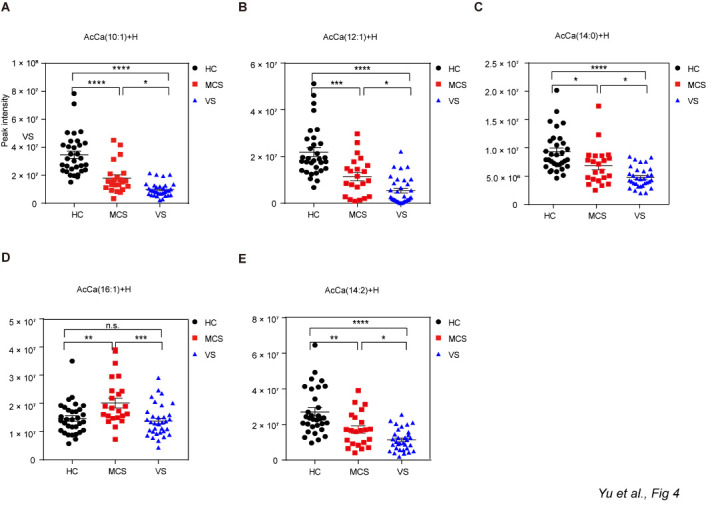
Different levels of AcCa among HC group, MCS group and VS group. Data represent as the mean ± SEM; *P<0.05, **P<0.01, ***P<0.001, ****P<0.0001, n.s.: no significant difference, one-way ANOVA. AcCa: acetylcarnitine; HC: healthy controls; VS: vegetative state; MCS: minimally conscious state; HC: healthy controls; VS: vegetative state; MCS: minimally conscious state.

Moreover, compared to the HC group, the top differential metabolites based on VIP score in the EMCS group showed a substantial difference from those of the DoC groups (Fig. 4A-B). Only 13 metabolites (Spermine, L-Tyrosine, Pyruvic acid, L-Cystine, Adenine, Taurine, D-Glucose, Pyridoxamine, Xanthosine, Cytidine monophosphate, Oxidized glutathione, Imidazole and 4-Hydroxyproline) were identified among the top differential metabolites in both comparisons (HC-MCS-VS groups and HC-EMCS-AD groups).

### Untargeted lipidomic analysis of serum from VS and MCS patients and HCs

Lipids are the essential molecules in the metabolism of the human body [25], but little is known about lipid metabolism in VS and MCS patients. We further expanded the sample size for lipidomic analysis.

Serum samples of the 86 subjects were analyzed with an unbiased approach using a UPLC-Orbitrap™ MS/MS. In total, 1536 species and 32 classes of lipid molecules were identified by the positive- and negative-ion models (Fig. 5A). Compared with the HC group, there were significant differences among 20 classes of lipids in the VS and MCS groups (Fig. 5B-F, Supplementary Fig. 3-4). Specifically, the mean levels of most lipids were lower in the VS and MCS groups, although the zymosterol level was higher in each of the VS and MCS groups (Fig. 5E). A prominent separation trend was found between the VS and HC groups for principal component 2. Similar to the findings of the PCA model of the metabolomics, a prominent separation trend was found among the three groups for principal component 2 (Fig. 5G). The data for the MCS group were also intermediate between the HC and VS groups although closer to that of the VS group. These findings are in agreement with our grouping based on CRS-R scores (HC group, MCS group and VS group), thus validating the reliability of these results.

**Figure 7.**
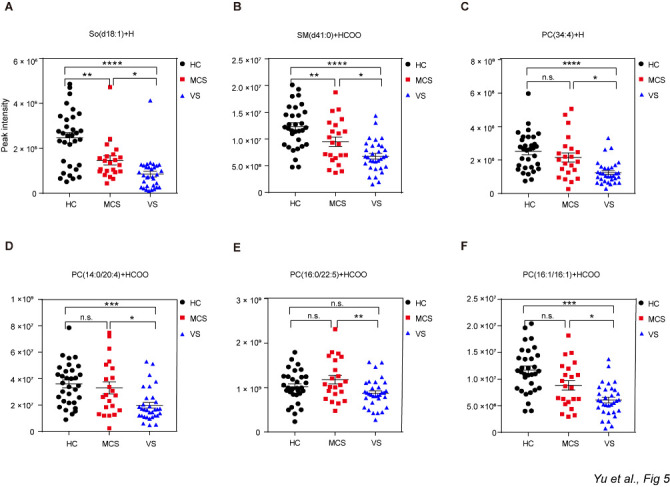
Different levels of So, PC and SM among the HC, MCS and VS groups. Data represent as the mean ± SEM; *P<0.05, **P<0.01, ***P<0.001, ****P<0.0001, n.s.: no significant difference, one-way ANOVA. PC: phosphatidylcholine; SM: sphingomyelin; So: sphingosine; HC: healthy controls; VS: vegetative state; MCS: minimally conscious state.

Further analyses revealed that the serum levels of 74 lipids differed significantly between the VS and MCS groups (Student’s t-test, *P*<0.05;). Among these 74 lipids, the differences for 11 were the most dramatic (*P*<0.01; Supplementary Table 2). Interestingly, all 11 lipids were significantly lower in the VS group compared with the MCS group when the three groups were compared together (one-way ANOVA, *P*<0.05). These lipids included five acetylcarnitine (AcCa) congeners (Fig. 6A-F), one sphingosine (Fig. 7A), one sphingomyelin (Fig. 7B), and four phosphatidylcholine (PC) (Fig. 7C-F).

**Figure 8.**
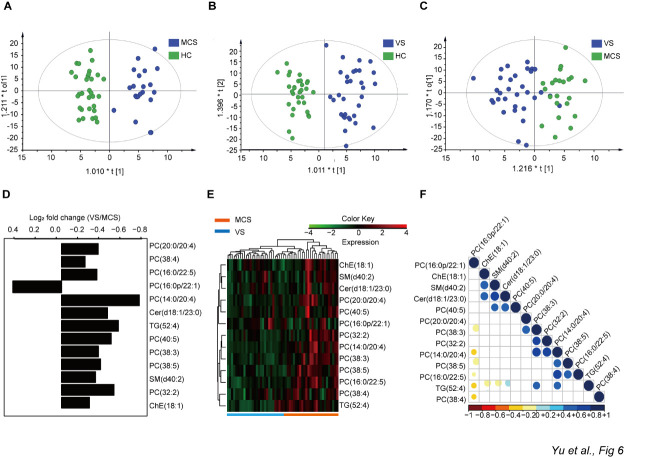
Multivariate analysis and univariate analysis of lipids detected by untargeted lipidomics. A) Orthogonal partial least squares-discriminant analysis (OPLS-DA) score plot between the HC and MCS groups. B) OPLS-DA score plot between the HC and VS groups. C) OPLS-DA score plot between the VS and MCS groups. D) Fold change in the serum levels of various lipids in the VS group compared with the MCS group. E) Hierarchical clustering analysis indicating natural variations in lipid levels in the VS and MCS groups. F) Heat map that correlated with lipids in the VS and MCS groups; HC: healthy controls; VS: vegetative state; MCS: minimally conscious state.

To determine which lipids were unique to each group and identify unique lipids as potential biomarkers to distinguish VS patients from MCS patients, the data were analyzed with an OPLS-DA model (Fig. 8A-C). Similar to the findings of the PCA model, a clear separation was apparent between each of the two groups using the predictive principal component t1. Comparisons between any two groups were performed using multivariate and univariate statistical significance criteria (VIP > 1, *P*<0.05). We identified 13 lipids in the VS and MCS groups (12 downregulated and 1 upregulated in the VS group) for which the levels differed significantly from those of the HC group (Fig. 8D). To validate these data and understand their correlation with observed differences in the biological states of the VS and MCS groups, a hierarchical clustering analysis and correlation analysis were performed (Fig. 8E-F). These analyses revealed that the VS and MCS groups were significantly distinguished according to the intensity of these 13 lipids, suggesting that these lipids may be used as biomarkers to diagnose VS and MCS and/or distinguish VS from MCS patients.

### Targeted lipidomic analysis of serum obtained from VS and MCS patients and HCs

**Figure 9.**
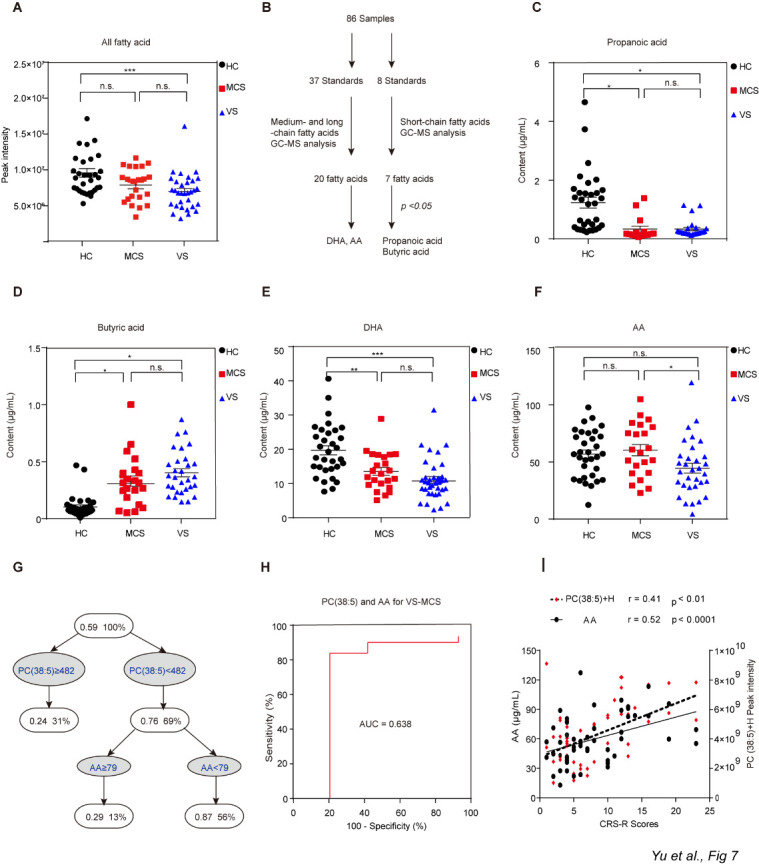
Analyses of free fatty acids and identification of candidate biomarkers. A) Comparison of free fatty acid levels detected by untargeted lipidomics in the HC, VS and MCS groups. Data represent as the mean ± SEM; *P<0.05, ***P<0.001, one-way ANOVA. B) Experimental flow for obtaining the results from targeted lipidomics. C-F) Difference analysis for free fatty acids detected by targeted lipidomics between the VS and MCS groups and the HC group. Data represent as the mean ± SEM; *P<0.05, **P<0.01, ****P<0.0001, n.s.: no significant difference, one-way ANOVA. DHA: docosahexaenoic acid; AA: arachidonic acid. G) Decision tree for predicting differences in certain lipid species and their levels between VS and MCS. H) ROC curves for PC(38:5)-H and AA for use in distinguishing VS patients from MCS patients. AUC, area under the receiver-operating characteristic curve. I) Level of PC(38:5)-H and AA correlated to CRS-R scores. CRS-R: Coma Recovery Scale-Revised; HC: healthy controls; VS: vegetative state; MCS: minimally conscious state.

Owing to the relatively low sensitivity of untargeted lipidomics, the levels of free fatty acids detected by untargeted lipidomics were lower in the VS group than in the HC group (Fig. 9A), but no difference was found between any other two groups (MCS & HC, VS & MCS). Therefore, we carried out targeted lipidomic measurements to quantify free fatty acids in serum using GC-MS for further analysis (Fig. 9B).

The mass spectra yielded 27 peaks that matched those observed in the standard samples. Each fatty acid was identified based on retention time and then quantified. Compared with the HC group, the levels of propanoic acid, butyric acid and docosahexenoic acid (DHA) were significantly lower in both the VS and MCS groups (Fig. 9C-F). The level of arachidonic (AA) was significantly lower in the VS group compared with the MCS group, but no such difference was apparent between the VS and HC groups or the MCS and HC groups (Fig. 9F), suggesting that AA level may be able to distinguish between patients with VS or MCS.

### Identification of lipid biomarkers to distinguish VS patients from MCS patients

Using untargeted lipidomics, we identified 13 lipids for which the levels differed significantly between the VS and MCS groups (Supplementary Table 3). We also used targeted lipidomics and identify AA for which the level differed significantly between these two groups. To determine whether these lipids and free fatty acids could be used as potential biomarkers to distinguish between VS and MCS patients, we first assessed their diagnostic performance individually using ROC analysis. Unfortunately, none of the individual candidate biomarkers yielded sufficient accuracy (Supplementary Table 4). Next, we established a diagnostic panel of 14 lipids (13 identified by untargeted lipidomics and AA identified by targeted lipidomics) that could distinguish VS from MCS patients and then conducted logistic regression and decision tree analyses (Fig. 9G). Interestingly, PC(38:5)-H together with AA yielded excellent diagnostic power. Subsequent decision tree analysis revealed that PC(38:5)-H≥482 or AA≥79 indicated MCS, whereas PC(38:5)-H<482 and AA<79 indicated VS.

To further validate the ability of PC(38:5)-H and AA to distinguish MCS from VS patients, we conducted leave-one-subject-out cross-validation. Cross-validation worked well in the leave-one-subject-out context, with an area under the curve equal to 0.638 (*P*=0.088, 95% CI: 0.463-0.813) and standard error of cross-validation equal to 0.089. We then assessed the predictive ability of the model based on the ROC curve (Fig. 9H), which yielded 77.8% accuracy, 78.1% sensitivity, and 77.3% specificity when the optimal cutoff point was set as 0.1425. Notably, the serum abundances of PC(38:5)-H and AA correlated positively with CRS-R scores, which is the gold standard for diagnosing and distinguishing VS and MCS patients (Fig. 9I); the respective correlation coefficients were 0.52 (*P*<0.0001) and 0.41 (*P*<0.01). Together, these results strongly suggested that PC(38:5)-H and AA may be used as potential clinical biomarkers to help diagnose and distinguish between patients with VS and MCS.

## DISCUSSION

To the best of our knowledge, this study is the first to comprehensively investigate metabolic profiles, including metabolism profiles in plasma and lipid profiles in serum, from patients with disorders of consciousness [1]. The metabolomics and lipidomics analysis detected 137 metabolites and 1536 lipids and 27 free fatty acids. We identified not only metabolites and lipids significantly altered in the VS/MCS groups compared with the HC group but also those differentially regulated between the VS and MCS groups. More importantly, we demonstrated that PC(38:5)-H combined with AA may be used as a potential biomarker set to aid in the diagnosis/distinction of VS and MCS with high accuracy, but further investigator is required.

The patients that we selected had suffered TBIs more than 6 months prior on average (195.2 ± 136.1 days). Thus, their states of consciousness were stable. Severe brain injury changed the metabolic profiles of their plasma. The differences we observed in the metabolic profiles are able to distinguish between levels of DoC. The metabolites enriched in plasma of TBI patients may be involved in the restoration of consciousness. Six metabolites were identified as having levels that gradually increased across the HC, MCS and VS groups, whereas two metabolites were decreased in patients with DoC compared to normal subjects, which suggested that altering these abnormal metabolites might be potentially beneficial for the patients with DoC. However, there were no statistically significant differences between the MCS and VS groups. A larger sample size will be necessary for further investigation in the future.

We also carried out a global metabolic pathway analysis and observed that purine metabolism pathway was the most impacted pathway that differed between the DoC (MCS and VS) and HC groups. The purine metabolism pathway is involved in nearly all the biochemical processes related to nucleic acid metabolism. Metabolites in the purine metabolism pathway not only play crucial roles in DNA and RNA synthesis, but also function in multiple physiological processes including inflammation [26] and neuronal differentiation [27]. In our study, the purine metabolism pathway was significantly down-regulated in VS/MCS patients relative to healthy controls, suggesting the collapse of energy production (exhaustion of AMP and ATP), which is a major cause of brain cell death [28]. Accordingly, the alteration of purine metabolism might be one of the harmful responses to TBIs that are experienced by DoC patients. Further investigation into this possibility is needed.

All patients in our studies suffered DoC as a result of sTBI. Our studies were designed to avoid potential influence from metabolomic differences of other etiologies, providing less heterogeneous results for the specific mechanism underlying metabolic abnormalities in patients with different levels of consciousness. In addition, we included a non-DoC neurological disease control (i.e., AD) and patients who recovered from DoC (i.e., EMCS). We observed that patients with AD showed quite different metabolic profiles from those in the EMCS group, suggesting that metabolic abnormalities in different types of brain damage indeed differ in this regard. Moreover, compared to the HC group, only 13 of the top differential metabolites based on VIP score in the EMCS group overlapped with those in the DoC groups, suggesting that metabolic abnormalities in TBI patients with or without DoC are not the same, even if they have a similar course.

The contribution of altered lipid metabolism to the pathogenesis of traumatic brain injury has been well studied [29, 30]. In particular, it has been demonstrated that the mean concentrations of PCs, SM, DGs and free fatty acids increase following traumatic brain injury[29, 31]. Moreover, traumatic brain injury perturbs brain energy metabolism[32-34], which completely normalizes 7 days later[35]. Lipid metabolism in chronic DoC, however, remains largely unclear. In our study, we found an overall decrease in the levels of lipids in the VS and MCS groups. Specifically, almost all classes of lipids were significantly decreased in these two groups compared with the HC group. The amount of energy supplied by lipid oxidation is important for maintaining cellular homeostasis consciousness [36]. Combined with previous metabolomics result that purine metabolism pathway was significantly downregulated in VS group and MCS group compared to HC group, we hypothesized that an imbalance in energy metabolism may play an important role in the pathophysiology of DoC. Interestingly, of the lipid subclasses we analyzed, zymosterol was dramatically increased in the VS/MCS groups. Zymosterol is an important metabolite for the biosynthesis of steroids and vitamin D2/D3 [37]. Long-term bedridden patients who lack sun exposure, which may lead to the accumulation of precursors of zymosterol [38], may have enhanced levels of zymosterol. In addition, the fact that zymosterol production is stimulated by enhanced levels of TNF-α in the brain [39] suggests that perturbation of zymosterol level might be related to oxidative stress and lipid peroxidation in patients with chronic DoC. Notably, among 20 subclasses, only AcCa level was lower in the VS group compared with the MCS group, suggesting that this lipid may be relevant to the pathophysiological processes of DoC.

One of our most interesting findings is that the serum levels of 11 lipids differed significantly between the VS and MCS groups, and this information has not been reported previously. Notably, almost half of these lipids were found to be congeners of AcCa. AcCa is naturally produced by neurons and is mainly involved in modulating brain energy and maintaining mitochondrial homoeostasis [40, 41]. In our study, the level of AcCa was significantly lower in the VS group compared with the MCS group, suggesting that disorders of energy metabolism caused by mitochondrial dysfunction may be a pathological mechanism of DoC. Moreover, AcCa mediates certain critical physiological aspects of brain function, including the production of neurotrophic factors and neurohormones, synaptic morphology, and synaptic transmission mediated by several neurotransmitters [40]. Recently, AcCa was also found to be efficacious for the treatment of certain neurological diseases [42, 43], suggesting that it may also have efficacy for treatment of DoC.

Another important highlight of our study is that we identified serum biomarkers to distinguish between VS and MCS patients. In a 14-lipid panel, PC(38:5)-H together with AA yielded >77% accuracy, sensitivity, and specificity for the diagnosis of VS and MCS and for distinguishing VS patients from MCS patients. This is a very preliminary study for us to distinguish VS and MCS patients with serum markers with metabolomic/lipidomic analysis. Accurately differentiating between patients with VS and those with MCS is vital for prescribing appropriate treatment. Currently, a diagnosis of VS or MCS relies mainly on CRS-R. This method, however, lacks sufficient accuracy. For example, a large number of studies have shown that up to 43% of patients who ultimately are diagnosed as VS are initially misdiagnosed based on their CRS-R score alone [44, 45]. Although repeated CRS-R assessments within a short period can help improve the accuracy of diagnosis for patients with DoC, the rate of misdiagnosis is not significantly reduced[46]. In addition, electrophysiology and imaging also can improve the accuracy of diagnosis [47-49]. These studies, however, suffered from a relatively small number of patients. Economic concerns and other considerations such as the poor spatial resolution of electrophysiology may prevent the widespread use of these methods for diagnosis. Moreover, the type/severity of injury as well as the brain region(s) affected can also affect the accuracy of CRS-R-based diagnosis. Serum lipid markers identified in this work, either alone or in combination with other methods, could potentially be used to enhance the accuracy of diagnosis for VS and MCS. Once optimized, this approach could possibly be used as a supporting method alongside CRS-R scores. In addition, perturbations in blood PC levels have been linked to DoC [50]. For example, administration of citicoline or CDP-choline, which are PC precursors, significantly improves electroencephalographic tracing and alleviates consciousness impairment after head injury [51, 52]. Similar to PC, AA also participates in the molecular and cellular pathways of consciousness [53].

This study has several limitations. First, though the patients in this study received uniform conventional therapy, we did not exclude the effect of some medications on their plasma metabolomic profiling, such as different kinds of antibiotics. The influence of differing diets among patients was also uncontrollable. In addition, the sample size of our metabolomics analysis is relatively small and there was no crossover between the patients who were in the metabolomic profiling and lipidomic profiling groups. Third, the up-regulated and down-regulated pathways we found among those groups cannot be further verified *in vivo* in the laboratory because there is currently no reliable animal model for chronic DoC. Fourth, in Table S4, the area under the curves of the lipids that we selected was around 0.7, but their specificity and sensitivity were not ideal. We combined PC(38:5)-H and arachidonic acid for diagnosis using decision tree and leave-one-subject-out cross-validation. Although this produced >77% accuracy, sensitivity, and specificity for the diagnosis of VS and MCS, the leave-one-subject-out cross-validation is just an internal validation and a larger cohort should be investigated for external validation.

## Supplementary Materials

The Supplemenantry data can be found online at: www.aginganddisease.org/EN/10.14336/AD.2020.0812.


