# Supplementary material for: Metabolic Abnormalities in Patients with Chronic Disorders of Consciousness
Source: Aging Dis. 2021 Apr 1;12(2):386–403. doi: 10.14336/AD.2020.0812 (PMC7990357; doi:10.14336/AD.2020.0812)
Supplement: Supplementary file 1 [file AD-12-2-386-s.pdf]

SUPPLEMENTARY DATA

Metabolic Abnormalities in Patients with Chronic Disorders of Consciousness

Jie Yu[1,#], Fanxia Meng[1,#] Fangping He[1], Fei Chen[2], Wangxiao Bao[1], Yamei Yu[1], Jintao Zhou[1], Jian Gao[3], Jingqi Li[3], Yao Yao[4], Woo-ping Ge[5*], Benyan Luo[1*]

© 2020. Yu J et al. Published online at http://www.aginganddisease.org/EN/10.14336/AD.2020.0812

SUPPLEMENTARY DATA

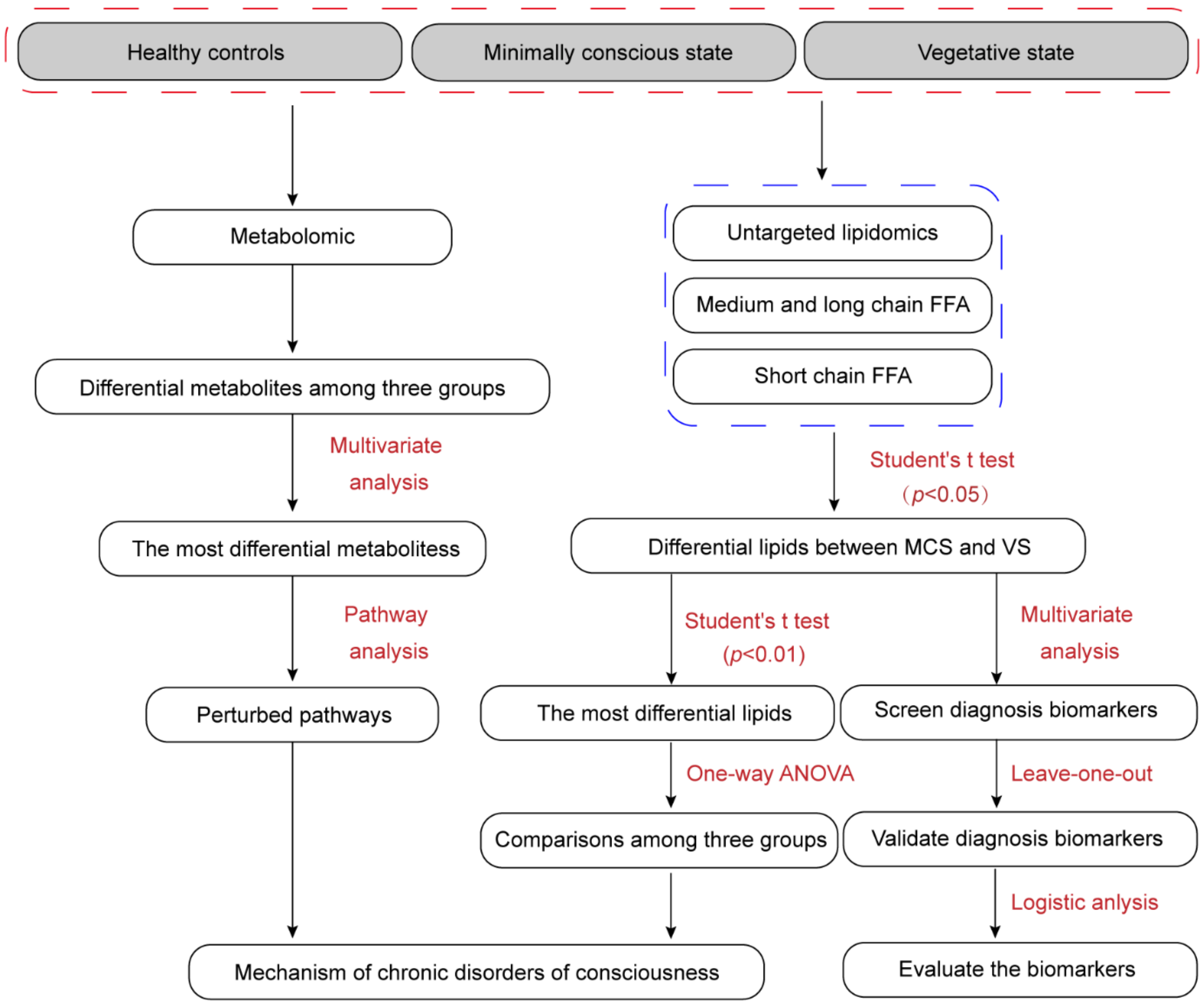

Supplementary Figure 1. **Flowchart of the experimental workflow for the study.** Healthy controls (HS), patients in vegetative state (VS) and minimally conscious state (MCS) were recruited for metabolomic and lipidomic studies. Both metabolite and lipid change specific to different levels of consciousness were examined. Potential lipids or metabolites that are responsible for distinguishing VS and MCS groups were identified in our analysis. FFA: free fatty acid. VS: vegetative state; MCS: minimally conscious state.

© 2020. Yu J et al. Published online at http://www.aginganddisease.org/EN/10.14336/AD.2020.0812

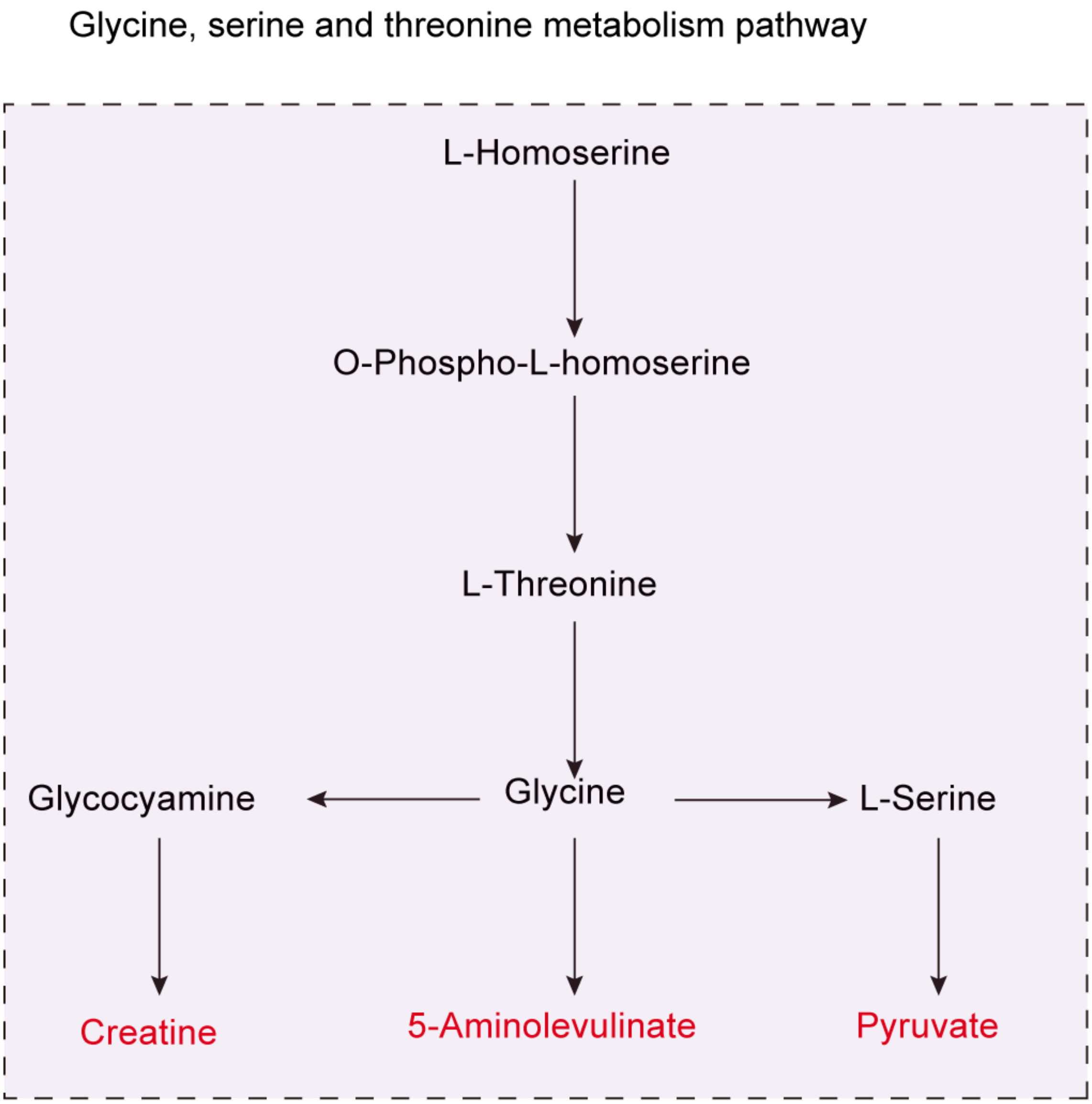

Supplementary Figure 2. Glycine, serine and threonine metabolism pathways in HC, MCS, and VS groups. Metabolites highlighted in red color were the ones that increased in these pathways in MCS and VS groups compared to HC group. HC: healthy controls; VS: vegetative state; MCS: minimally conscious state.

© 2020. Yu J et al. Published online at http://www.aginganddisease.org/EN/10.14336/AD.2020.0812

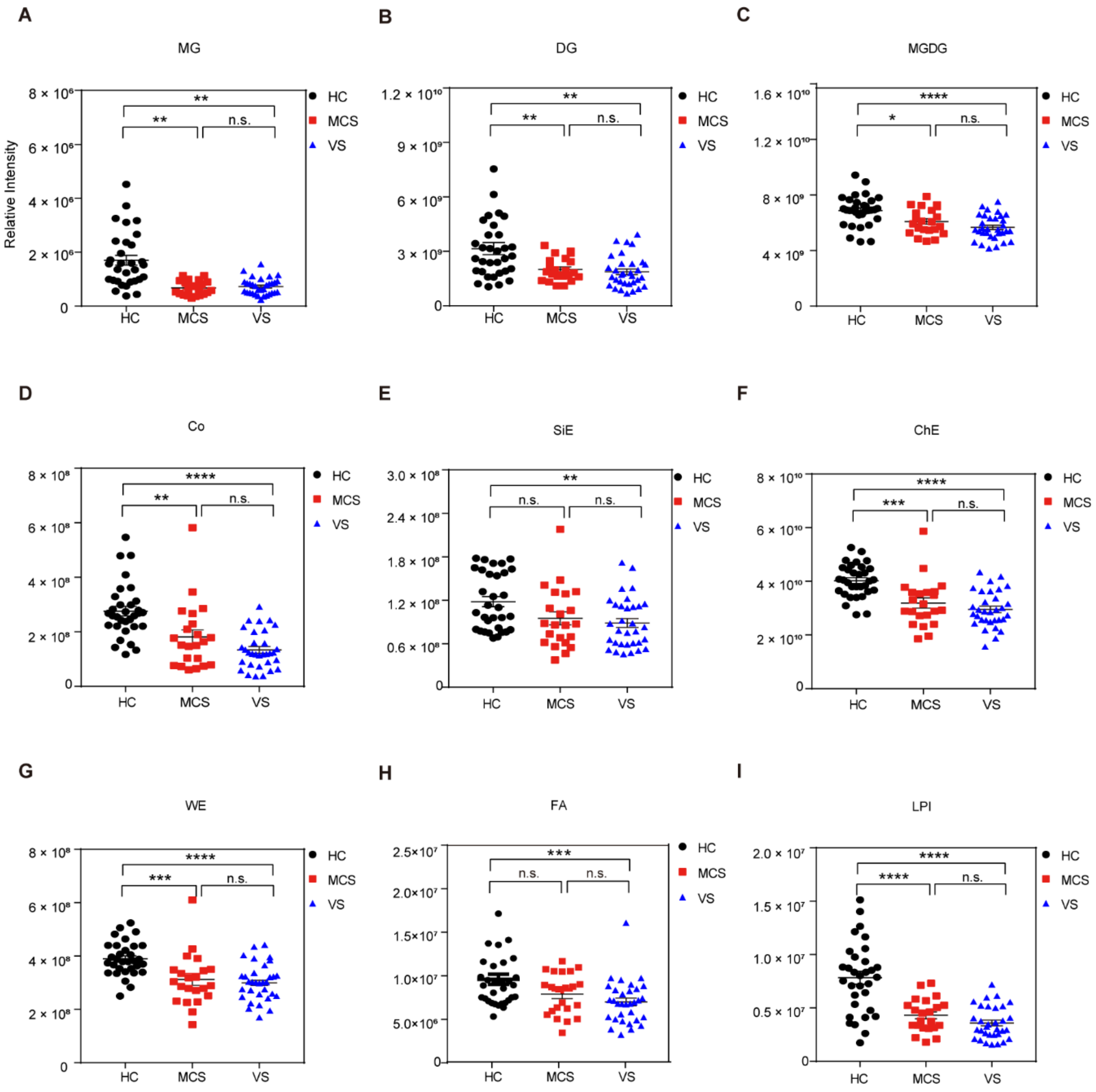

Supplementary Figure 3. Different levels of lipid subclasses among the HC, MCS and VS groups. Data represent as the mean ± SEM; **P*<0.05, ***P*<0.01, ****P*<0.001, *****P*<0.0001, n.s.: no significant difference, one-way ANOVA. PE: phosphatidylethanolamine; PS: phosphatidylserine; PG: phosphatidylglycerol; PI: phosphatidylinositol; Cer: ceramides; CerG1: simple glc series; HC: healthy controls; VS: vegetative state; MCS: minimally conscious state.

© 2020. Yu J et al. Published online at http://www.aginganddisease.org/EN/10.14336/AD.2020.0812

SUPPLEMENTARY DATA

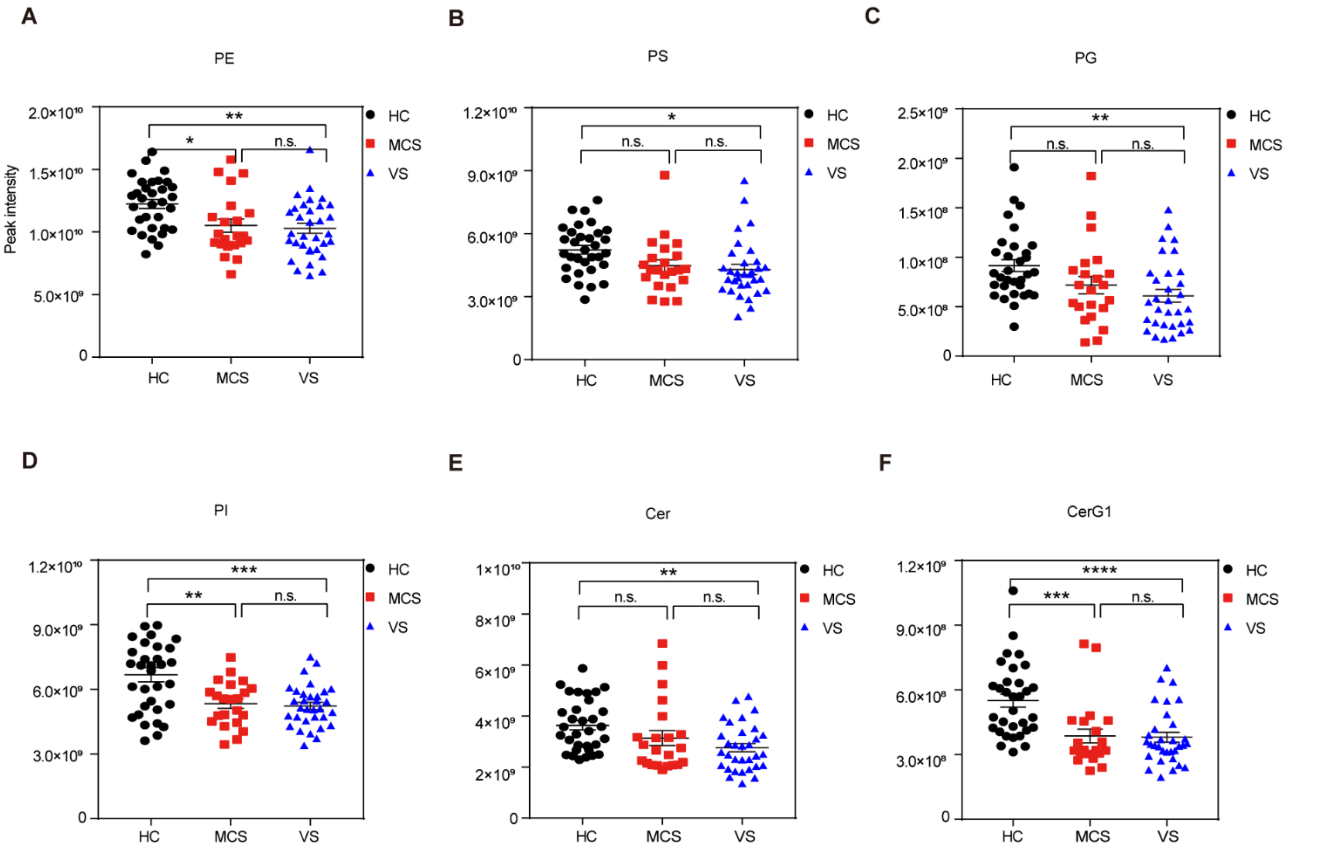

Supplementary Figure 4. Different levels of lipid subclasses among the HC, MCS and VS groups. Data represent as the mean ± SEM; **P*<0.05, ***P*<0.01, ****P*<0.001, *****P*<0.0001, n.s.: no significant difference, one-way ANOVA. MG: monoglyceride; DG: diglyceride; MGDG: monogalactosyldiacylglycerol; Co: coenzyme; SiE: sitosterol ester; ChE: cholesterol ester; WE: wax esters; FA: fatty acid; LPI: lysophosphatidylinositol; HC: healthy controls; VS: vegetative state; MCS: minimally conscious state.

Supplementary Table 1. Clinical characteristics of AD patients, EMCS patients and healthy controls.

Metabonomics				*P* value		
Characteristics	HCs	AD	EMCS	HCs-EMCS	HCs-AD	EMCS-AD
Patients(n)	6	19	15	/	/	/
Male/Female(n)	2/4	11/8	10/5	0.163	0.294	0.601
Age(years)	55.8 ± 4.8	70.5 ± 8.3	55.1 ± 8.7	0.639	<0.001	<0.001
GCS	/	/	7.2±1.3	/	/	/
CRS-R	/	/	21.9	/	/	/
Cause	/	/	TBI	/	/	/

Continuous variables are expressed as the mean ± standard deviation (SD); TBI: traumatic brain injury; GCS: Glasgow Coma Scale; CRS-R: Coma Recovery Scale-Revised scores; HCs: healthy controls; AD: Alzheimer's disease; EMCS: Emerged from Minimally Conscious State.

© 2020. Yu J et al. Published online at http://www.aginganddisease.org/EN/10.14336/AD.2020.0812

SUPPLEMENTARY DATA

Supplementary Table 2. List of putatively identified lipid molecules differing between MCS and VS groups. The eleven lipids in the table are those with significant differences between these two groups (*P*<0.01, Student's *t*-test).

Lipids	Class	RT (min)	Fold change	*P* value
AcCa(10:1)+H	AcCa	1.613	0.550	0.0005
AcCa(16:1)+H	AcCa	2.568	0.681	0.0012
PC(34:4)+H	PC	10.237	0.579	0.0013
AcCa(12:1)+H	AcCa	1.820	0.473	0.0024
PC(16:1/16:1)+HCOO	PC	10.455	0.613	0.0045
PC(16:0/22:5)+HCOO	PC	11.439	0.794	0.0053
AcCa(14:0)+H	AcCa	2.408	0.699	0.0054
PC(14:0/20:4)+HCOO	PC	10.248	0.601	0.0062
SM(d41:0)+HCOO	SM	15.524	0.715	0.0069
AcCa(14:2)+H	AcCa	1.881	0.662	0.0094
So(d18:1)+H	So	3.023	0.582	0.0099

RT: Retention time; AcCa: acylcarnitine; PC: phosphatidylcholine; SM: sphingomyelin; So: sphingosine. VS: vegetative state; MCS: minimally conscious state

Supplementary Table 3. List of putatively identified lipid molecules differing between VS and MCS groups based on VIP >1 (PLSDA) and *P* <0.05 (Student's *t*-test).

Lipids	Class	RT (min)	Fold change	*P* value	VIP
PC(14:0/20:4)+HCOO	PC	23.608	0.858	0.026	8.407
ChE(18:1)+NH_4	ChE	10.439	0.690	0.017	4.498
Cer(d18:1/23:0)+HCOO	Cer	13.519	0.794	0.005	4.168
PC(38:5)+H	PC	11.727	0.775	0.035	3.016
PC(16:0/22:5)+HCOO	PC	13.107	0.801	0.014	2.703
SM(d40:2)+H	SM	12.747	0.786	0.046	2.126
PC(18:0/20:4)+HCOO	PC	21.796	0.833	0.044	1.669
PC(38:3)+H	PC	16.800	0.723	0.031	1.539
PC(32:2)+H	PC	10.248	0.787	0.025	1.473
PC(16:0p/22:1)+HCOO	PC	14.933	1.376	0.014	1.280
PC(40:5)+H	PC	11.439	0.741	0.028	1.218
PC(20:0/20:4)+HCOO	PC	12.385	0.601	0.006	1.217
TG(16:0/18:1/18:3)+NH_4	TG	13.091	0.710	0.025	1.151

RT: Retention time; VIP: variable importance in the projection; VS: vegetative state; MCS: minimally conscious state

Supplementary Table 4. The diagnostic performance of the lipids in 14-lipid panel.

Lipids name	AUC	Specificity	Sensitivity	*P* value	95%CI
ChE(18:1)-NH_4	0.609	0.219	1.000	0.175	0.454- 0.765
PC(32:2)-H	0.669	0.781	0.591	0.036	0.513- 0.825
SM(d40:2)-H	0.676	0.875	0.455	0.029	0.525- 0.827
PC(38:5)-H	0.690	0.875	0.591	0.018	0.536- 0.844
PC(38:3)-H	0.690	0.500	0.909	0.018	0.548- 0.833
PC(40:5)-H	0.682	0.438	0.909	0.024	0.539- 0.824
TG(16:0/18:1/18:3)-NH_4	0.696	0.625	0.773	0.015	0.555- 0.838
Cer(d18:1/23:0)-HCOO	0.663	0.375	0.909	0.043	0.517- 0.810
PC(14:0/20:4)-HCOO	0.702	0.813	0.591	0.012	0.553- 0.850
PC(16:0p/22:1)-HCOO	0.695	0.844	0.591	0.016	0.545- 0.844
PC(16:0/22:5)-HCOO	0.706	0.750	0.636	0.011	0.558- 0.854
PC(18:0/20:4)-HCOO	0.697	0.875	0.500	0.014	0.551- 0.844
PC(20:0/20:4)-HCOO	0.648	0.344	0.955	0.067	0.499- 0.797
Arachidonic acid	0.701	0.778	0.565	0.010	0.564- 0.837

AUC: area under the ROC curve; ROC: Receiver-Operating Characteristic.

SUPPLEMENTARY DATA

Supplementary Table 5. The cause of each patient with VS, MCS or EMCS.

No.	State	Gender	Age	Cause
**Lipidomics**				
1	MCS-13	Male	51	Fall
2	MCS-56	Male	70	Traffic accident
3	MCS-72	Male	35	Traffic accident
4	MCS-80	Female	49	Traffic accident
5	MCS-98	Female	53	Traffic accident
6	MCS-104	Female	60	Fall
7	MCS-110	Male	21	Traffic accident
8	MCS-134	Male	44	Traffic accident
9	MCS-157	Female	51	Traffic accident
10	MCS-158	Male	60	Traffic accident
11	MCS-162	Female	56	Fall
12	MCS-166	Male	56	Traffic accident
13	MCS-167	Male	68	Traffic accident
14	MCS-170	Male	44	Fall
15	MCS-171	Male	31	Traffic accident
16	MCS-180	Female	40	Traffic accident
17	MCS-199	Female	65	Traffic accident
18	MCS-201	Male	55	Fall
19	MCS-202	Female	59	Fall
20	MCS-221	Female	58	Traffic accident
21	MCS-222	Male	53	Traffic accident
22	MCS-227	Female	70	Traffic accident
23	VS04	Male	64	Fall
24	VS12	Female	49	Traffic accident
25	VS14	Male	68	Fall
26	VS28	Male	63	Traffic accident
27	VS35	Male	27	Traffic accident
28	VS53	Male	67	Traffic accident
29	VS59	Male	44	Traffic accident
30	VS66	Male	55	Fall
31	VS67	Male	23	Traffic accident
32	VS68	Female	55	Traffic accident
33	VS78	Female	49	Traffic accident
34	VS81	Male	30	Fall
35	VS99	Female	43	Traffic accident
36	VS107	Female	24	Traffic accident
37	VS133	Male	21	Traffic accident
38	VS154	Female	53	Traffic accident
39	VS155	Male	43	Traffic accident
40	VS160	Male	52	Traffic accident
41	VS161	Female	67	Traffic accident
42	VS163	Male	62	Traffic accident
43	VS164	Male	42	Fall
44	VS165	Male	49	Traffic accident
45	VS168	Male	16	Traffic accident
46	VS187	Male	60	Traffic accident
47	VS189	Male	67	Traffic accident
48	VS200	Male	52	Traffic accident
49	VS214	Male	35	Traffic accident
50	VS220	Male	72	Traffic accident
51	VS226	Male	49	Fall
52	VS228	Male	55	Traffic accident
53	VS237	Female	68	Fall
54	VS244	Male	44	Traffic accident
**Metabolomics**				
1	VS1	Male	43	Traffic accident
2	MCS2	Male	43	Traffic accident
3	VS3	Female	63	Traffic accident

© 2020. Yu J et al. Published online at http://www.aginganddisease.org/EN/10.14336/AD.2020.0812

4	VS4	Female	63	Traffic accident
5	VS5	Female	63	Traffic accident
6	VS6	Male	56	Traffic accident
7	VS7	Male	64	Fall
8	VS8	Male	69	Traffic accident
9	MCS9	Male	70	Traffic accident
10	MCS10	Male	70	Traffic accident
11	VS11	Male	77	Fall
12	VS13	Male	77	Fall
13	MCS14	Male	61	Fall
14	MCS15	Male	66	Traffic accident
15	MCS16	Male	66	Traffic accident
16	MCS17	Female	63	Traffic accident
17	MCS18	Female	63	Traffic accident
18	MCS19	Female	54	Traffic accident
19	VS20	Male	68	Fall
20	VS21	Male	68	Fall
21	VS22	Male	68	Fall
22	MCS23	Female	61	Traffic accident
23	MCS24	Female	61	Traffic accident
24	EMCS5	Male	44	Traffic accident
25	EMCS14	Male	58	Traffic accident
26	EMCS23	Male	69	Traffic accident
27	EMCS25	Female	55	Fall
28	EMCS36	Male	53	Traffic accident
29	EMCS39	Female	50	Fall
30	EMCS43	Female	54	Traffic accident
31	EMCS301	Male	60	Traffic accident
32	EMCS203	Female	46	Traffic accident
33	EMCS209	Female	46	Traffic accident
34	EMCS210	Male	57	Fall
35	EMCS4	Male	45	Traffic accident
36	EMCS110	Male	69	Fall
37	EMCS207	Male	69	Traffic accident
38	EMCS3	Male	51	Fall

VS: vegetative state; MCS: minimally conscious state; EMCS: emerged from a minimally conscious state.

© 2020. Yu J et al. Published online at http://www.aginganddisease.org/EN/10.14336/AD.2020.0812
